# Correlated Expression of *HMGA2* and *PLAG1* in Thyroid Tumors, Uterine Leiomyomas and Experimental Models

**DOI:** 10.1371/journal.pone.0088126

**Published:** 2014-02-07

**Authors:** Markus Klemke, Marietta Henrike Müller, Werner Wosniok, Dominique Nadine Markowski, Rolf Nimzyk, Burkhard Maria Helmke, Jörn Bullerdiek

**Affiliations:** 1 Center for Human Genetics, University of Bremen, Bremen, Germany; 2 Institute of Statistics, University of Bremen, Bremen, Germany; 3 Institute of Pathology, University of Heidelberg, Heidelberg, Germany; 4 Institute for Medical Genetics, University of Rostock, University Medicine, Rostock, Germany; Universite Libre de Bruxelles (ULB), Belgium

## Abstract

In pleomorphic adenomas of the salivary glands (PASG) recurrent chromosomal rearrangements affecting either 8q12 or 12q14∼15 lead to an overexpression of the genes of the genuine transcription factor PLAG1 or the architectural transcription factor HMGA2, respectively. Both genes are also affected by recurrent chromosomal rearrangements in benign adipocytic tumors as e. g. lipomas and lipoblastomas. Herein, we observed a strong correlation between the expression of *HMGA2* and *PLAG1* in 14 benign and 23 malignant thyroid tumors. To address the question if *PLAG1* can be activated by HMGA2, the expression of both genes was quantified in 32 uterine leiomyomas 17 of which exhibited an overexpression of *HMGA2*. All leiomyomas with *HMGA2* overexpression also revealed an activation of *PLAG1* in the absence of detectable chromosome 8 abnormalities affecting the *PLAG1* locus. To further investigate if the overexpression of *PLAG1* is inducible by HMGA2 alone, *HMGA2* was transiently overexpressed in MCF-7 cells. An increased *PLAG1* expression was observed 24 and 48 h after transfection. Likewise, stimulation of *HMGA2* by FGF1 in adipose tissue-derived stem cells led to a simultaneous increase of *PLAG1* mRNA. Altogether, these data suggest that *HMGA2* is an upstream activator of *PLAG1*. Accordingly, this may explain the formation of tumors as similar as lipomas and lipoblastomas resulting from an activation of either of both genes by chromosomal rearrangements.

## Introduction

The DNA-binding protein high mobility group AT-hook 2 (HMGA2) and the zinc finger protein PLAG1 share a common role in the molecular pathogenesis of certain benign tumors, e. g. of the salivary glands and of adipose tissue. Pleomorphic adenomas are benign tumors of myoepithelial origin most often located in the parotid glands. Based on the existence of clonal chromosomal aberrations cytogenetic subtypes of pleomorphic adenomas can be distinguished [1]–[3]. Of these, structural rearrangements involving chromosomal regions 8q12 and 12q14∼15 are most frequently observed. Both types of aberrations seem to occur mutually exclusive [2], [4]–[6] and appear to be causally linked to the development of the disease. Though independently related to the same histologic tumor entity, the target genes rearranged by these aberrations encode proteins with different functions. *HMGA2* is located within the region 12q14∼15 which is frequently affected by chromosomal alterations [7]–[9] and encodes a DNA-binding non-histone protein mainly expressed during embryogenesis and in embryonic as well as in adult stem cells [10]–[16]. *PLAG1* (Pleomorphic adenoma gene 1) mapping to 8q12 encodes a genuine transcription factor encompassing seven zinc finger domains and a carboxyterminal transactivation domain. *PLAG1* is developmentally regulated and highly expressed in certain fetal tissues [17], [18]. Oncogenic activation of *PLAG1* plays a key role in the development of lipoblastomas [19], hepatoblastomas [20], chronic lymphocytic leukemia [21] as well as in pediatric gastro-intestinal stromal tumors [22]. PLAG1 has been found to bind the insulin-like growth factor gene (*IGF-II*) promoter and to stimulate its activity [23]–[25].

Similar but not identical to what is seen in pleomorphic adenomas both genes participate in the genesis of benign adipose tissue tumors. Chromosomal translocations affecting 12q14∼15 and targeting *HMGA2* are a common finding in lipomas often as a t(3;12)(q27;q14∼15) [26], [27]. In contrast, translocations of 8q12 are a recurrent cytogenetic deviation in lipoblastomas, i. e. rare benign adipose tissue tumors of early childhood [19], [28]–[30]. Interestingly, pleomorphic adenomas and lipoblastomas share the most frequent type of this rearrangement, i.e. a simple reciprocal translocation t(3;8)(p21;q12). Recently, an infantile lipoblastoma with rearrangements of the *HMGA2* locus has been described as well [31]. These findings raise the question why transcriptional activation of either of these two genes leads to the formation of tumors as similar as lipomas and lipoblastomas. One likely explanation is that they both act as part of a common pathway. Besides pleomorphic adenomas and adipose tissue tumors, another link between these two genes has recently emerged: in thyroid tumors, the expression level of *HMGA2* has been found to allow a good discrimination between benign and malignant thyroid lesions [32]–[35]. Likewise, Prasad et al. have recently studied the genome-wide mRNA expression patterns of benign and malignant thyroid tumors in a systematic approach aimed at the identification of those genes best suited to distinguish between both types of thyroid lesions. The expression of *HMGA2* ranked at the first position followed by *Kallikrein 7* (*KLK7*), *Mannose receptor, C type 2* (*MRC2*), *Leucine-rich repeat kinase 2* (*LRRK2*), and *PLAG1* [36].

Because of the apparent relationship of *HMGA2* and *PLAG1* in the molecular pathogenesis of salivary gland adenomas and adipose tissue tumors, we also quantified and compared the expression of *HMGA2* and *PLAG1* mRNA in thyroid adenomas as well as in papillary and follicular thyroid carcinomas. To further analyze the relationship between these two genes, we also quantified the *PLAG1* expression in 32 uterine leiomyomas (UL) with as well as without 12q14 rearrangements. In addition, the *PLAG1* expression was quantified in adipose tissue-derived stem cells (ADSCs) upon a stimulation of *HMGA2* by FGF1. Furthermore, the MCF-7 breast cancer cell line, which has previously been used as a model in transfection experiments aiming at the functions of HMG proteins [37], [38], was transiently transfected with a eukaryotic expression vector encoding for wild-type HMGA2 to evaluate whether *PLAG1* can be transcriptionally activated by HMGA2.

## Methods

### Tissue Samples

Formalin-fixed, paraffin-embedded tissue samples of 37 thyroid tumors were classified histologically. Fourteen cases were classified as follicular adenomas (FA), eleven tumors were diagnosed as papillary carcinomas (PTC), and four of them were follicular variants (FV PTC). The remaining twelve tumors were follicular thyroid carcinomas (FTC). Cryopreserved tissue samples of 32 uterine leiomyomas that have been analyzed cytogenetically [16], [39]–[41], were also used for quantification of gene expression. The karyotypes of the leiomyomas are given in supplementary Table S1. Karyotype description followed ISCN 2009 [42].

### RNA Isolation and Reverse Transcription

In case of thyroid tumors, RNA was isolated from six 5 µm sections of FFPE tissues with the RNeasy FFPE kit (Qiagen, Hilden, Germany). RNA from cryopreserved leiomyoma tissues as well as from cultivated cells (ADSCs and MCF-7 cell line) was isolated with the RNeasy Mini kit (Qiagen). Of each sample, 250 ng RNA was used for reverse transcription with M-MLV reverse transcriptase and random hexamers (Invitrogen, Darmstadt, Germany) according to the manufacturer’s instructions.

### Quantitative Real-time RT-PCR (qRT-PCR)

Quantitative real-time RT-PCR was performed with the TaqMan Universal PCR Master Mix on a 7300 Real-Time PCR System (Applied Biosystems) as described elsewhere [32].

The relative quantification of the *HMGA2* expression was performed with the *HMGA2*-specific TaqMan assay Hs00171569_m1 (Applied Biosystems, Darmstadt, Germany). For the detection of the endogenous control *HPRT1*, primers 5′-GGC AGT ATA ATC CAA AGA TGG TCA A-3′ and 5′-GTC TGG CTT ATA TCC AAC ACT TCG T-3′ were used in combination with the *HPRT1*-specific hydrolysis probe 6FAM-CAA GCT TGC TGG TGA AAA GGA CCC C-TAMRA. The *PLAG1* expression was quantified with the TaqMan assay Hs00231236_m1 (Applied Biosystems). Reaction conditions were as follows: 2 min at 50°C and 10 min at 95°C followed by 50 cycles of 15 sec at 95°C and 1 min at 60°C.

### Stimulation of *HMGA2* Expression

Human adipose tissue-derived stem cells were obtained and treated as described previously [16]. For stimulation with fibroblast growth factor 1 (FGF1), cells were plated at a density of 3×10^5^ cells/9.6-cm dish. After 24 hours the serum concentration was reduced to 1%. Another 24 hours later the medium was replaced by serum-free medium supplemented with 25 ng/ml of human recombinant FGF1 (Jena Bioscience, Jena, Germany). Twelve, 24, and 72 hours after growth factor addition, cells were harvested and total RNA was extracted. As controls cells were cultured in medium 199 supplemented with 1% fetal bovine serum (FBS) without FGF1.

### Plasmid DNA Purification

Plasmid pCR3.1 containing the sequence coding for human wild type HMGA2 as well as the empty vector serving as a control (for generation of the vectors see [43]) were purified using the NucleoBond Maxi Plus EF Kit (Macherey-Nagel, Düren, Germany) according to the manufacturer’s instructions.

### Cell Culture and Transfection of MCF-7 Cells

The cell line MCF-7 (breast cancer) was maintained in medium 199 (Life Technologies) supplemented with 20% FBS in an incubator at 37°C and 5% CO_2_. When grown till confluence, cells were passaged using TrypLE Express (Life Technologies). The day before transfection, 150,000 cells were seeded in 6-well plates (Nunc, Wiesbaden, Germany) in 2 ml medium 199 supplemented with 20% FBS and allowed to attach for 24 h. Transfection complexes were prepared in a total volume of 500 µl in serum free medium 199 without antibiotics, 2.5 µg of the respective plasmid DNA and 7 µl Lipofectamine LTX transfection reagent (Life Technologies). A mock control treated with transfection reagent only as well as a non-treated control were included for each incubation period. During the formation of transfection complexes (25 min at room temperature), old growth medium was replaced by 2 ml fresh medium. Transfection complexes were then pipetted drop-wise to the cells. After incubation for 24 h or 48 h, cells were harvested in 350 µl RLT buffer (Qiagen) containing β-mercaptoethanol for RNA isolation using the RNeasy Mini Kit and Qiacube (Qiagen). As a positive control, the expression of *IGF2BP2* (synonym: *IMP2*), which is known to be regulated by HMGA2 [44], [45], was quantified by qRT-PCR as described above using the TaqMan Assay Hs00538956_m1 (Applied Biosystems).

### Statistical Analysis

As a measure of association between the relative *HMGA2* and *PLAG1* expression levels, Spearman’s rank correlation coefficient was calculated using the data of all 37 thyroid tumor samples analyzed. In addition, Pearson’s correlation coefficient was calculated using the relative expression values as well as the ΔC_T_ values. In case of uterine leiomyomas, the expression levels of both *HMGA2* and *PLAG1* were classified as high or low based on the ΔC_T_ values (*HMGA2*: <0/≥0, *PLAG1*: <3/≥3). The agreement between the *HMGA2* and the *PLAG1* classes was then quantified and tested by the kappa coefficient of agreement.

### Ethics Statement

This study was conducted in accordance with the Declaration of Helsinki [46] and the guidelines of the German National Ethics Council [47]. In case of uterine leiomyomas, the use of tissue samples taken from surgically removed tumors was approved by the Ethics Committee of the Ärztekammer Bremen. Written informed consent was obtained from all patients. For the investigations on thyroid tumors, archival samples originally taken for diagnostic purposes were used. All thyroid tumor samples were used in previous studies [32], [48].

## Results

### Expression of *HMGA2* and *PLAG1* in Adenomas and Carcinomas of the Thyroid

**Figure 1:**
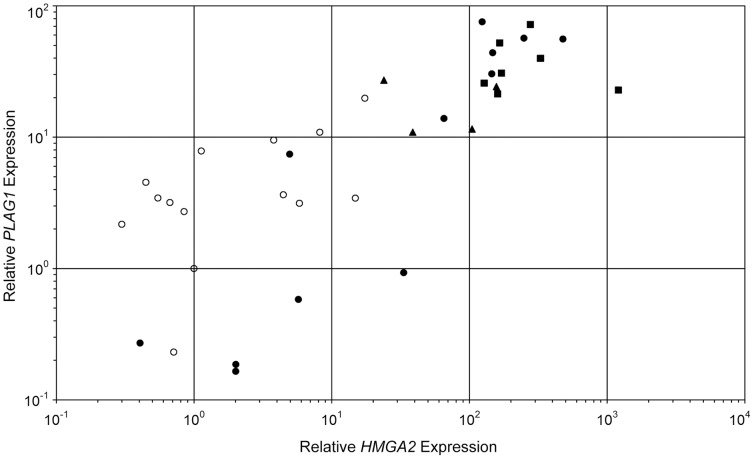
Relative expression of *PLAG1* versus *HMGA2* in 37 thyroid tumors. The calibrator sample for the quantification of both genes’ expression is a follicular adenoma. Open circle: follicular adenoma; full circle: follicular carcinoma; square: papillary carcinoma; triangle: follicular variant of papillary carcinoma.

The expression of *HMGA2* and *PLAG1* was quantified in 37 thyroid tumors. An adenoma with low expression of both genes was chosen as calibrator for the relative quantification. The highest relative *HMGA2* expression observed in 14 follicular adenomas was 17.4, whereas the expression levels in four FV PTC ranged between 23.9 and 156.9. Even higher expression levels ranging between 128.2 and 1207.5 were observed in seven PTC (Fig. 1).

**Figure 2:**
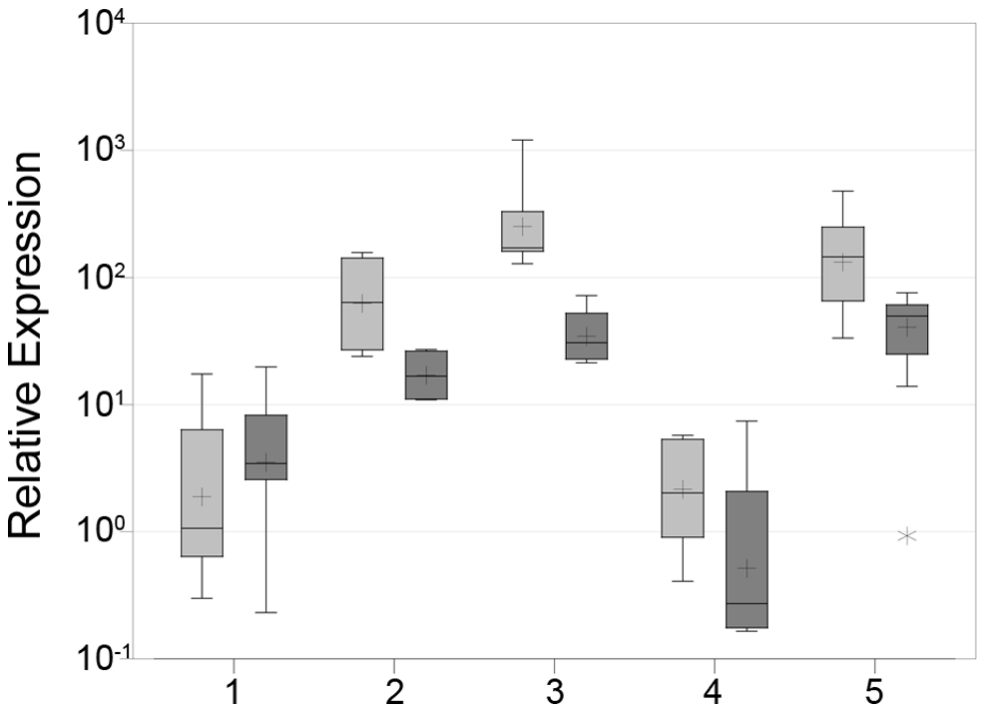
*HMGA2* expression in thyroid tumors. Box-whisker-plot indicating the relative expression of *HMGA2* (light gray) and *PLAG1* (dark gray) in five groups of thyroid tumors. Group 1: follicular adenomas (n = 14), group 2: follicular variants of papillary carcinomas (n = 4), group 3: papillary carcinomas (n = 7), group 4: follicular thyroid carcinomas without *HMGA2* over-expression (n = 5), group 5: follicular thyroid carcinomas with *HMGA2* overexpression (n = 7). The asterisk marks an outlier.

Based on their *HMGA2* expression, two groups of follicular thyroid carcinomas can be distinguished. Seven cases clearly overexpressed *HMGA2* with expression levels ranging between 33.3 and 478.3, whereas in five FTCs the *HMGA2* expression was within the range of adenomas (0.4 up to 5.73, Fig. 2).

The relative expression of *PLAG1* ranged between 0.2 and 19.8 in follicular adenomas and was below ten in twelve of 14 cases (Figs. 1 and 2). In the follicular variants of papillary carcinomas, it ranged between 10.9 and 27.2, and in the classic variants of papillary carcinomas, the *PLAG1* expression ranged between 21.3 and 72.2. Akin to *HMGA2*, *PLAG1* is also expressed at higher levels in papillary carcinomas. In the subgroup of follicular carcinomas without or with only slight *HMGA2* overexpression, the *PLAG1* expression levels ranged between 0.3 and 0.6 in four cases while one case showed a slight overexpression (7.4). In contrast, *PLAG1* was expressed at levels between 13.9 and 55.9 in six follicular carcinomas overexpressing *HMGA2*. No overexpression of *PLAG1* (0.9) was detected in only one FTC with high *HMGA2* expression.

Thus, as to the expression level of both genes a strong variation was noted even within the group of malignant tumors. If an alternative overexpression of either gene represents different pathways of tumorigenesis one would expect no or even an inverse correlation. On the other hand a correlation would indicate their involvement in just one pathway. Spearman’s correlation coefficient thus was calculated as a measure of association between the *HMGA2* and *PLAG1* expression for all 37 tumor samples and resulted in a high value (ρ = 0.82, p<0.0001) indicating that the expression of both genes is strongly correlated in thyroid tumors. For reasons of comparison, Pearson’s correlation coefficient was calculated and also indicates a strong correlation when using the ΔC_T_ values (r = 0.77, p<0.0001). Due to the exponential transformation, the linear relationship is less pronounced on the basis of relative gene expression values (r = 0.44, p<0.001).

### Expression of *HMGA2* and *PLAG1* in Uterine Leiomyomas

Recurrent rearrangements affecting the chromosomal band 12q14 are known to cause an overexpression of *HMGA2* in uterine leiomyomas. Therefore, UL (n = 32) were chosen to study a presumable effect of *HMGA2* on the *PLAG1* expression. One myoma with a normal 46,XX karyotype was chosen as calibrator for the qRT-PCR data obtained for both genes.

Of 17 UL with known 12q14 rearrangement including case 646 without cytogenetically visible breakpoint but rearranged *HMGA2* locus according to FISH [40], an overexpression of *HMGA2* was detected in 16 cases. In the remaining case without elevated *HMGA2* expression despite a cytogenetically visible t(12;14)(q15;q24) (no. 503) FISH revealed no rearrangement of the *HMGA2* locus [39].

Only 1/15 UL without cytogenetically detectable 12q14 aberration also showed an elevated *HMGA2* expression. A hidden rearrangement of *HMGA2* was suspected but no evidence for such a rearrangement was obtained by FISH (case 520).

**Figure 3:**
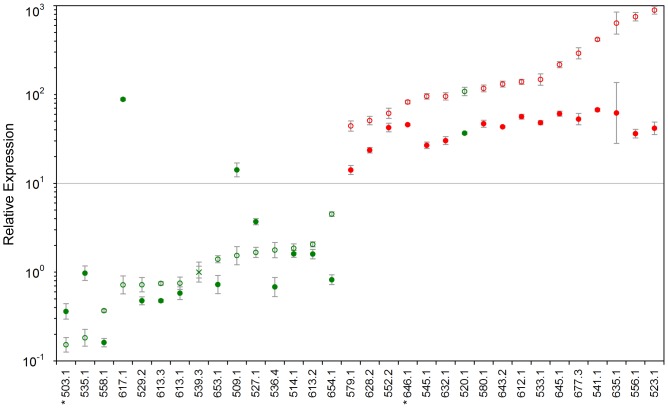
Relative expression of *HMGA2* and *PLAG1* in uterine leiomyomas. Open circle: *HMGA2*, full circle: *PLAG1*, cross: calibrator sample. Green: the *HMGA2* locus is not affected by rearrangement (n = 16), red: the *HMGA2* locus is affected by rearrangement (n = 16). In two cases marked with an asterisk, cytogenetic and FISH results differ. A t(12;14)(q15;q24) was visible in case 503, but FISH excluded a rearrangement of *HMGA2*. In case 646 FISH revealed a hidden rearrangement of *HMGA2* [40].

The expression of *PLAG1* was clearly elevated in all 17 UL which overexpress *HMGA2*. In 15 UL with low *HMGA2* expression, elevated levels of *PLAG1* were detected in only two cases (509 and 617, Fig. 3). Spearman’s rank correlation coefficient was calculated for the expression of both genes and resulted in a high value (ρ = 0.75, p<0.0001). Pearson’s correlation coefficient also indicated a significant correlation when it was calculated based on the ΔC_T_ values (r = 0.85, p<0.0001). Similarly a high and significant degree of agreement was found between the low/high expression classification of samples obtained by both parameters (kappa = 0.8735, 95% CI = (0.752, 1.000), see also Table 1).

**Table 1:** Case numbers of uterine leiomyomas assigned to high vs. low gene expression classes according to ΔC_T_ values.

*HMGA2* expression	*PLAG1* expression	
	Low (ΔC_T_ ≥3)	High (ΔC_T_ <3)
**Low (ΔC_T_ ≥0)**	n = 13	n = 2
**High (ΔC_T_ <0)**	n = 0	n = 17

### Stimulation of *HMGA2* Expression in ADSCs

**Figure 4:**
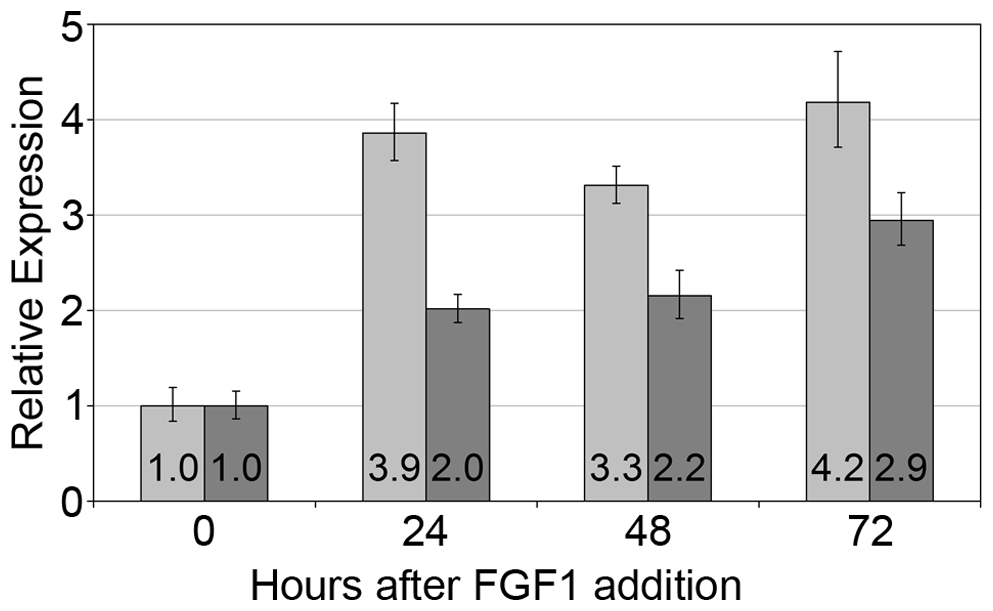
Relative expression of *HMGA2* and *PLAG1* in ADSCs after stimulation with FGF1. Light gray: *HMGA2*, dark grey: *PLAG1*. Expression levels were measured before and 24, 48 and 72 h after stimulation with the FGF1 protein.

To stimulate the expression of *HMGA2* in ADSCs, FGF1 was chosen because it is a known inducer of *HMGA2* [49]. The relative quantification revealed a 3.9-fold increase of the *HMGA2* mRNA level 24 h after the addition of FGF1. Simultaneously, the *PLAG1* expression increased by 2-fold (Fig. 4). After 48 h, the *HMGA2* level decreased only slightly, but a clear peak could not be identified, because the highest level was observed after 72 h with a 4.2-fold increase. The expression of *PLAG1* increased continuously to a 2.9-fold expression 72 h after the addition of FGF1.

### Transient Transfection of the MCF-7 Breast Cancer Cell Line

**Figure 5:**
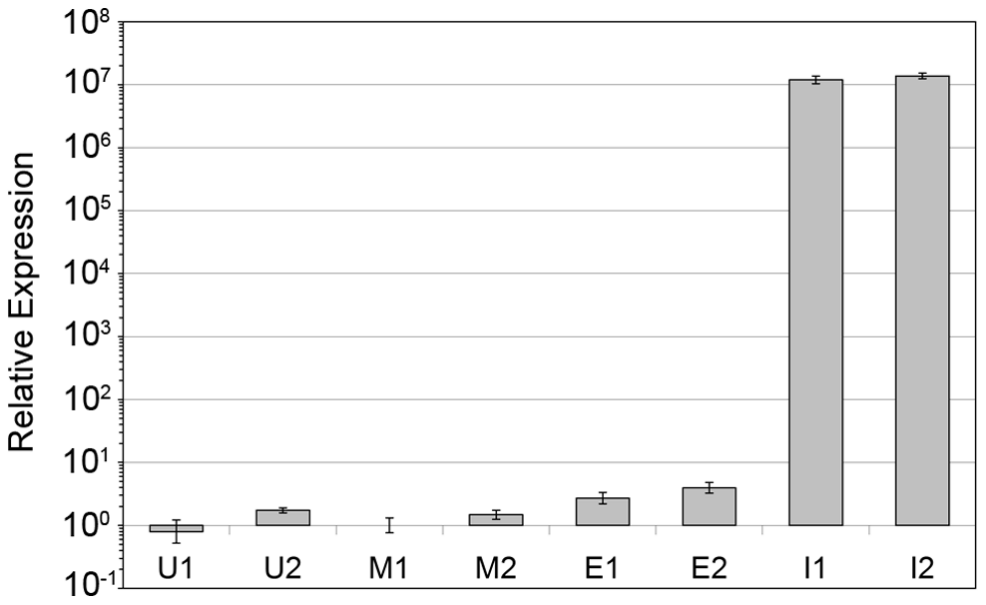
Relative expression of *HMGA2* in transiently transfected MCF-7 cells. The gene expression was measured 24(1) and 48 h (2) after transfection. U: untreated cells, M: mock-transfected cells (transfection reagent only), E: cells transfected with the empty expression vector, I: cells transfected with the *HMGA2* expression vector.

**Figure 6:**
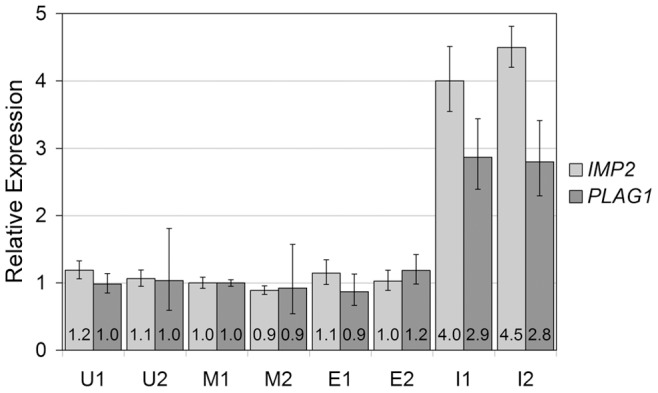
Relative expression of *IMP2* and *PLAG1* in MCF-7 cells transfected with an *HMGA2* expression vector. Light gray: *IMP2*, dark grey: *PLAG1*. The gene expression was measured 24 h (1) and 48 h (2) after transfection. The expression level of mock-transfected cells 24 h after transfection was used as calibrator. U: untreated cells, M: mock-transfected cells (transfection reagent only), E: cells transfected with the empty expression vector, I: cells transfected with the expression vector containing the insert encoding for HMGA2.

The expression of *HMGA2* in MCF-7 cells was quantified 24 and 48 h after transfection in mock transfected cells and in cells transfected either with the empty vector or with the vector containing an insert encoding for wild-type HMGA2. qRT-PCR revealed a strongly increased *HMGA2* expression in the latter (Fig. 5) indicating successful transfection. In addition, the expression of *IMP2* encoding for the insulin-like growth factor 2 mRNA binding protein 2 was quantified, because it is a known target of *HMGA2* [44], [45]. Whereas no effect was apparent upon transfection with the empty vector, *IMP2* was clearly upregulated upon transfection with the vector encoding for HMGA2. Quantifications at both 24 and 48 h after transfection resulted in a similar value of at least 4-fold *IMP2* overexpression (Fig. 6). Similar results were obtained for *PLAG1*, the expression of which remained unaltered by the addition of transfection reagent alone (“mock”) as well as after transfection with the empty vector. Cells transfected with the vector containing the *HMGA2* insert, however, showed an approximately 2.8-fold increase in *PLAG1* expression as compared to mock transfected cells 24 h as well as 48 h after transfection (Fig. 6).

## Discussion

Previous studies on pleomorphic adenomas of the salivary glands have shown that *PLAG1* is frequently overexpressed in PASG with normal karyotype as well as with 12q14∼15 abnormalities [50], [51].

Akin to what has been described for PASG, the results of the present study indicate that both genes are co-expressed in thyroid tumors as well as in leiomyomas. In papillary carcinomas (including follicular variants), both genes are expressed at higher levels than in follicular adenomas. Follicular carcinomas with high *HMGA2* expression levels also express *PLAG1* at elevated levels.

A correlation between chromosomal rearrangements affecting the *HMGA2* locus and the *HMGA2* protein expression has been shown in uterine leiomyomas [52]. Moreover, it has been shown that in thyroid carcinomas the increased expression of *HMGA2* and *PLAG1* is detectable on the mRNA as well as on the protein level [53]. Therefore, the correlation of *HMGA2* and *PLAG1* mRNA expression described herein is expected to reflect a correlation at the protein level as well.

Besides the typical rearrangements involving chromosomal band 8q12 including the most frequent t(3;8)(p21;q12), an activation of *PLAG1* in pleomorphic adenomas of the salivary glands occurs also in tumors with 12q14∼15 abnormalities lacking 8q12 aberrations [50]. Besides 13/17 tumors with an apparently normal karyotype, 5/10 pleomorphic adenomas with 12q13∼15 abnormalities were found to overexpress *PLAG1*. In the same study, the *PLAG1* expression was investigated in three UL, and two cases were also found to overexpress *PLAG1*, but no cytogenetic data were available for these three tumors.

These findings suggest alternative mechanisms of *PLAG1* activation in tumorigenesis other than gene rearrangements. The results presented herein point to HMGA2 as an upstream regulator of *PLAG1* and are additionally confirmed by the correlation between the expressions of both genes in uterine leiomyomas. An activation of *HMGA2* in UL by 12q14 aberrations is well known [7], [39], [54]–[56]. Therefore, we chose 15 UL with an apparently normal karyotype or with chromosomal aberrations affecting regions other than 12q14 and 17 cases with 12q14 aberrations to quantify the expression of *PLAG1* and *HMGA2* simultaneously. Of the 15 UL showing low *HMGA2* levels, 13 also showed low levels of *PLAG1*. The two remaining cases showed an elevated *PLAG1* expression despite a low *HMGA2* mRNA expression, thus pointing to mechanisms other than *HMGA2* upregulation being responsible for *PLAG1* activation. In pleomorphic adenomas of the salivary gland cryptic, intrachromosomal 8q rearrangements have been observed leading to a fusion of *PLAG1* with *CHCHD7* or *TCEA1* [57]. Because the breakpoints are located in the 5′-noncoding regions of both fusion partners, these fusions lead to an activation of *PLAG1* by promoter swapping. Similar events that escape detection by conventional cytogenetics may have caused the upregulation of *PLAG1* observed in two UL without visible rearrangements affecting 8q12. In all 17 UL with elevated *HMGA2* levels a concomitant overexpression of *PLAG1* was noted. The fact that increased *HMGA2* levels were always linked to elevated *PLAG1* levels suggests HMGA2 as an activator of *PLAG1*. Because no chromosomal rearrangements affecting 8q12 were present in these 17 UL, we assumed that elevated HMGA2 levels caused by 12q14 abnormalities trigger the activation of *PLAG1*. In turn, HMGA2 may exert its stimulating effect on the growth of UL with 12q14 aberrations [54] at least in part by activating *PLAG1*, which was shown to possess transactivating capacity [18] and is able to activate downstream target genes like *IGF-II*.

The observation that FGF1-stimulated expression of *HMGA2* is accompanied by increased *PLAG1* expression levels (Fig. 4), further supports the hypothesis that HMGA2 exerts a *PLAG1*-activating function. Likewise, transient overexpression of *HMGA2* using an appropriate vector is sufficient to trigger an upregulation of *PLAG1*. Although factors beside *HMGA2* may be involved in the upregulation of *PLAG1*, comparable to the cooperation between HMGA2 and NF-κB in the transcriptional activation of the IFN-β gene *IFNB1* [58], [59] or *IMP2* [44], [45], the *HMGA2* expression vector alone was sufficient to exert an activating effect not only on the known *HMGA2* target *IMP2* but also on *PLAG1*.

In conclusion, it has been shown that *PLAG1* overexpression in thyroid carcinomas as well as in uterine leiomyomas with aberrations affecting the chromosomal region 12q14∼15 strongly correlates with the overexpression of *HMGA2*. The increased expression levels of both genes upon stimulation of ADSCs with FGF1 and particularly the upregulation of *PLAG1* upon introduction of an *HMGA2* expression vector into the MCF-7 cell line strongly suggest that *PLAG1* is regulated by HMGA2 and that histologic similarities observed between benign tumors with either rearrangements of the *HMGA2* or the *PLAG1* locus result from activation within the same pathway.

## Supporting Information

Table S1 Karyotypes of all 32 uterine leiomyomas.
(DOC)
