# Supplementary material for: Correlated Expression of HMGA2 and PLAG1 in Thyroid Tumors, Uterine Leiomyomas and Experimental Models
Source: PLoS One. 2014 Feb 7;9(2):e88126. doi: 10.1371/journal.pone.0088126 (PMC3917869; doi:10.1371/journal.pone.0088126)
Supplement: Table S1 — Karyotypes of all 32 uterine leiomyomas. (DOC) [file pone.0088126.s001.doc]

Supplementary Table S1: Karyotypes of all 32 uterine leiomyomas. ^1)^ *HMGA2* is unaffected by the rearrangement according to FISH ^[39]^. ^2)^ FISH revealed a hidden rearrangement of *HMGA2* ^[40]^.

| **Case** | **Karyotype** |
| --- | --- |
| 503.1 ^1)^ | 46,XX,inv(5)(q15q31~33),t(12;14)(q15;q24)[13] |
| 509.1 | 46,XX[14] |
| 514.1 | 46,XX[17] |
| 520.1 | 46,XX[7] |
| 523.1 | 45,XX,t(12;14)(q15;q24),der(14)t(12;14)(q15;q24),-22[8] |
| 527.1 | 46,XX[10] |
| 529.2 | 46,XX[14] |
| 533.1 | 46,XX,r(1),t(1;12;14)(p36.3;q14;q24)[19] |
| 535.1 | 47,XX,+10[2]/46,XX[10] |
| 536.4 | 46,XX[11] |
| 539.3 | 46,XX[9] |
| 541.1 | 46,XX,t(12;14)(q15;q24)[5]/46,XX[9] |
| 545.1 | 46,XX,t(12;14)(q15;q24)[9]/46,XX[3] |
| 552.2 | 46,XX,t(2;12)(q33;q13)[17] |
| 556.1 | 46,XX,t(3;5;12)(q23~25;p13~15;q13~15)[11]/45,XX,idem,-22[10] |
| 558.1 | 46,XX[13] |
| 579.1 | 46,XX,t(12;15;14)(q15;q26;q24)[20] |

Supplementary Table S1 (continued).

| **Case** | **Karyotype** |
| --- | --- |
| 580.1 | 46,XX,der(7)del(7)(p)del(7)(q),add(8)(q),add(10)(q),t(12;14)(q15;q24)[19] |
| 612.1 | 46,XX,t(12;14)(q15;q24)[13]/46,XX,der(1)r(1;2),t(12;14)(q15;q24)[4] |
| 613.1 | 46,XX[15] |
| 613.2 | 46,XX[15] |
| 613.3 | 46,XX[16] |
| 617.1 | 46,XX,der(1)del(1)(p22),der(3)?t(1;3)(p22;q?),der(5)del(5),der(12)t(12;?) (q24.3;?),-14,-20,+mar1,+mar2[6] |
| 628.2 | 46,XX,?ins(12;14)(q15;q31q24)[5]/46,XX[14] |
| 632.1 | 46,XX,t(12;14)(q15;q24)[12]/46,XX,del(4)(q31~q32),der(10),?t(10,14) (q24;q32),t(12;14)(q15;q24)[9] |
| 635.1 | 46,XX,der(10),del(12)(q13~q14)[18] |
| 643.2 | 46,XX,t(12;14)(q15;q24)[14] |
| 645.1 | 45,XX,r(1),der(13;14)(q10;q10)t(12;14)(q15;q24)[20]/44,XX,-1,der(13;14) (q10;q10)t(12;14)(q15;q24)[6] |
| 646.1 ^2)^ | 46,XX,t(2;12)(p21;p13)[11] |
| 653.1 | 46,XX[14] |
| 654.1 | 46,XX[8] |
| 677.3 | 46,XX,add(1)(p13),r(1)(?p36.3q25),add(7)(q22),der(10)t(1;10)(q25;q22), der(12)add(12)(p11.2)add(q12),add(13)(q12)[20]/46,XX[3] |
